# Case Report: A Case of Valproic Acid-Induced Hyperammonemic Encephalopathy Associated With the Initiation of Lithium: A Re-duplicable Finding

**DOI:** 10.3389/fpsyt.2022.875636

**Published:** 2022-05-02

**Authors:** Anna Levy, Etienne Very, François Montastruc, Philippe Birmes, Adeline Jullien, Louis Richaud

**Affiliations:** ^1^Department of Psychiatry, Psychotherapy, and Art therapy, University Hospital Toulouse (CHU Toulouse), Toulouse, France; ^2^Toulouse NeuroImaging Center, Universit9 de Toulouse, Inserm, UPS, Toulouse, France; ^3^Department of Medical and Clinical Pharmacology, Centre of PharmacoVigilance and Pharmacoepidemiology, Toulouse University Hospital, Faculty of Medicine, Toulouse, France; ^4^Department of Pharmacy, University Hospital Toulouse (CHU Toulouse), Paule de Viguier Hospital, Toulouse, France

**Keywords:** encephalopathy, hyperammonemia, lithium, interaction, valproate sodium, pharmacokinetic interaction

## Abstract

**Introduction:**

Hyperammonemic encephalopathy (HAE) is a serious adverse effect of valproate semisodium, which is facilitated by the potential for drug interaction. However, despite frequent co-prescription of valproate semisodium and lithium, the role of this combination in the occurrence of HAE has not been defined in the literature. This case report concerns the occurrence of HAE concomitant with the initiation of lithium in a 29-year-old patient who had been placed on valproate semisodium for a schizoaffective disorder.

**Case Report:**

Due to a relapse while on a combined antipsychotic and mood-stabilizing therapy (paliperidone palmitate and valproate semisodium), a cross-taper from valproate semisodium to lithium was proposed. The initiation of lithium was accompanied by an acute confusional syndrome, an elevated serum valproate level and hyperammonemia suggestive of drug-induced HAE. The discontinuation of lithium and reduction of valproate semisodium led to neurological improvement, until a recrudescence of psychiatric symptoms justified a rechallenge of the combination within the framework of a new cross-taper. As soon as Lithium was re-initiated, an increase in the serum valproate level and hyperammonemia were again noted.

**Discussion:**

The mechanisms of valproate-related HAE involve various metabolic pathways. In this case, exploration of the iatrogenic hypothesis focused on the imputability of concomitant cannabis use and co-prescriptions of benzodiazepines, antipsychotics, and in all likelihood, mood stabilizers.

**Conclusion:**

Therefore, this case study suggests that Lithium plays a role in serum valproate level elevation, and supports the hypothesis of an association between an elevated serum valproate level, hyperammonemia and reversible encephalopathy. A more in-depth pharmacokinetic exploration would provide a better understanding of the mechanisms of these interactions and support for the benefit-risk balance associated with this frequent co-prescription.

## Introduction

Valproic acid—marketed as valproate or valproate semisodium—is a non-barbiturate anticonvulsant drug and a GABAergic mood stabilizer. It is indicated in neurology for the management of focal or generalized epilepsy (1) and in psychiatry for the management of manic and mixed episodes in bipolar disorder, commonly employed in the course of combination treatments (2–5). In the valproate semisodium adverse effects (AE) profile, encephalopathy is described as “rare” (0.1–1%), generally transient and which regresses on discontinuation of treatment or dose reduction, but which could also lead to serious complications requiring intensive (hemodialysis) and/or resuscitative management (6, 7). While the mechanisms underlying the occurrence of these AEs is still not perfectly understood, many cases of hyperammonemic encephalopathy (HAE) have been reported in the literature (8, 9) through which various risk factors can be identified. One such risk factor is the combination of several drugs which could contribute to this AE by drug interaction (10). To date, none of these articles have mentioned the risk of iatrogenic HAE specifically associated with the co-prescription of lithium and valproate semisodium. However, this combination of drugs is common (11). Therefore, this case report concerns the concomitant occurrence of HAE with the initiation of lithium in a 29-year-old patient with schizoaffective disorder treated with valproate semisodium who was hospitalized for acute manic relapse with mood-incongruent psychotic features.

## Case Report

### Case History

*Somatically*, the patient had no significant medical or surgical history, and in particular no documented drug allergy or AE. He had grade 1 obesity (99 kg, 180 cm, BMI: 30.56), was an active smoker, and had a history of addiction (cannabis) from which he had been abstinent for several years.

*Psychiatrically*, the patient had a history of schizoaffective disorder diagnosed at the age of 19 (illness duration of 10 years) according to DSM5 criteria (12), in combination with antisocial personality disorder and substance use disorder. Illness recurrences came in the form of acute manic psychosis, which were often triggered by a discontinuation of treatment and required multiple psychiatric hospitalizations (5 within the past 2 years), the most recent of which were by involuntary care.

*Therapeutically*, the initiation of long-acting injectables (LAI) in the form of Risperidone extended-release (ER) (50 mg/14 days) first of all ensured an initial period of clinical stability. However, 2 years before the onset of the current episode, a decompensation while on a single antipsychotic agent had motivated the initiation of a mood stabilizer (Valproate semisodium, 2,000 mg/d, with serum valproate concentration of 62 mg/L, within the therapeutic range of 41–100 mg/L) combined with antipsychotic treatment with paliperidone ER (100 mg/28 d). This dual therapy was then replaced by a combination of valproate semisodium (2,000 mg/day) and Risperidone (4 mg/d) in a context of absenteeism from outpatient follow-up. Six months prior to the onset of the current episode, dual therapy with valproate semisodium (2,000 mg/d) + paliperidone ER (100 mg/28 d) was re-initiated, with a well-tolerated dosage increase (to 2,500 mg/d and 150 mg/28 d, respectively, accompanied by an elevation in serum valproate levels from 67 mg/L before to 97 mg/L after drug dosage increase) without any further adjustment in the 4 months preceding this episode.

### Current Episode

After a short period of clinical stability however, a further interruption of both outpatient follow-up and drug treatment was accompanied by an increase in both manic (elated mood and psychomotor agitation) and psychotic symptoms (delusions of grandeur and persecution) leading to behavioral disturbances and hetero-aggressive threats against his neighbors. After police intervention, the patient was transported to the psychiatric emergency services for medical assessment, and hospitalized without consent in a psychiatric care unit, where further examinations were performed.

### During Hospitalization

Due to a relapse with a serum valproate level suggestive of drug non-compliance on admission (serological concentrations of valproate measured at 23 mg/l on admission, below the therapeutic range), the drug was re-prescribed at the usual doses and supplemented with diazepam, a benzodiazepine drug, and levomepromazine, a phenothiazine antipsychotic with tranquilizer properties. When clinical deterioration (acute manic psychosis with grandiose delusions and psychomotor agitation) again occurred under effective treatment [i.e., despite a serum valproate level within the therapeutic range (91 mg/l)], a change in mood stabilizer was proposed in an overlap-and-taper strategy, after pre-therapeutic assessment (with no outstanding features). Considering that the patient had many illness recurrences of manic polarity with psychotic symptoms, as well as a high metabolic syndrome risk valproate was replaced with lithium as mood stabilizer, which was initiated (day 0) at 400 mg/d. However, after the third administration of slow-release lithium salt, signs of fluctuating temporal disorientation signaled the onset of an acute confusional syndrome (d3). An iatrogenic etiology was immediately suspected, which led to the discontinuation of lithium the same day (d3) and the confusional syndrome was etiologically assessed.

### Diagnostic Assessment and Etiological Approach

Clinically, the patient presented with psychomotor retardation, disorders of alertness and global cognitive impairment [Mini-Mental State Examination (MMSE) score of 15/30 (13); Frontal Assessment Battery (FAB) score of 3/18 (14)] with no clinical signs on general or neurological examination). Biological examinations (complete blood count, electrolytes, liver, kidney, thyroid, coagulation and vitamin workup) revealed no significant abnormality (day 3). The brain computed tomography (CT) scan (day 5) showed nothing abnormal. The electroencephalogram (EEG) (d5) showed no seizure activity but presented a pattern indicating encephalopathy (fluctuating and slowing background activity with theta/alpha and delta waves, predominant in the posterior regions, reactive to verbal stimulation and with the change to eyes-open). Pharmacologically, serum lithium level measured 24 h after the third intake (day 3) showed subtherapeutic doses (undetectable <0.05 mmol/l; N: 0.8–1.2 mEq/l), whereas the serum valproate level was consistent with an overdose (130 mg/l) and was accompanied by a threefold elevation in serum ammonia level (182 μmol/l; N: 16–60). In addition, the blood toxicology screen showed cannabis use (THC-COOH: 16.9 ng/l) but did not suggest associated substance use.

### Diagnosis

The diagnosis of *drug-induced* hyperammonemic encephalopathy (HAE) was therefore proposed, and the valproate semisodium dose was decreased under clinical supervision (2,000 mg/d on day 4, 1,500 mg/d on day 6). The neurologist recommended no other urgent course of action.

### Course of the Condition

Dose reduction resulted in rapid neurological improvement, both clinically and on the electroencephalogram [check on day 7, i.e., 3 days *(steady-state*) after the first valproate semisodium dose reduction], associated with normalization of serum valproate (79 mg/l on day 7; 5 mg/l on day 10) and serum ammonia (66 μmol/l on day 7; 32 μmol/l on day 10) levels. However, regression of the encephalopathic condition quickly gave way to the reappearance of psychiatric symptoms due to the lack of effective treatment, which justified a re-increase in valproate semisodium dosage (2,500 mg/d on day 12, 5 days after the regression of the neurological disorders) and a monthly injection of paliperidone, which once again stabilized the patient without any clinical or biological adverse effect observed in 1 month (serum valproate level: 61 mg/l and serum ammonia level: 27 μmol/l on day 38, on valproate semisodium 2,500 mg/d).

### Rechallenge

After requesting an opinion from a pharmacology specialist—and in the absence of documentation in the literature regarding the possibility that lithium may have contributed to the picture presented, a change in mood stabilizer was again considered at day 46 (day 0') according to the same modalities. lithium was initiated at 400 mg/d, valproate semisodium was progressively decreased (2,000 mg/d on day 0', 1,500 mg/d on day 3' [...] discontinuation on day 12') and the patient was reassessed daily. No clinical adverse effects were reported. However, biologically, the results of the test done on day 6' showed an infratherapeutic serum lithium level (0.26 mEq/l with 400 mg/d of lithium) and a 6% increase in serum valproate level compared to the test done 2 weeks earlier (89 mg/l on day 6' compared to 61 mg/l on day 38), despite a 40% decrease in the valproate semisodium dose administered since the last test (1,500 mg/d on day 6' compared to 2,500 mg/d on day 38). In addition, this elevated serum valproate level was again associated with hyperammonemia (90 mg/l), with no proven consumption of toxic substances (negative urine toxicology screen).

### In Brief

**Figure 1:**
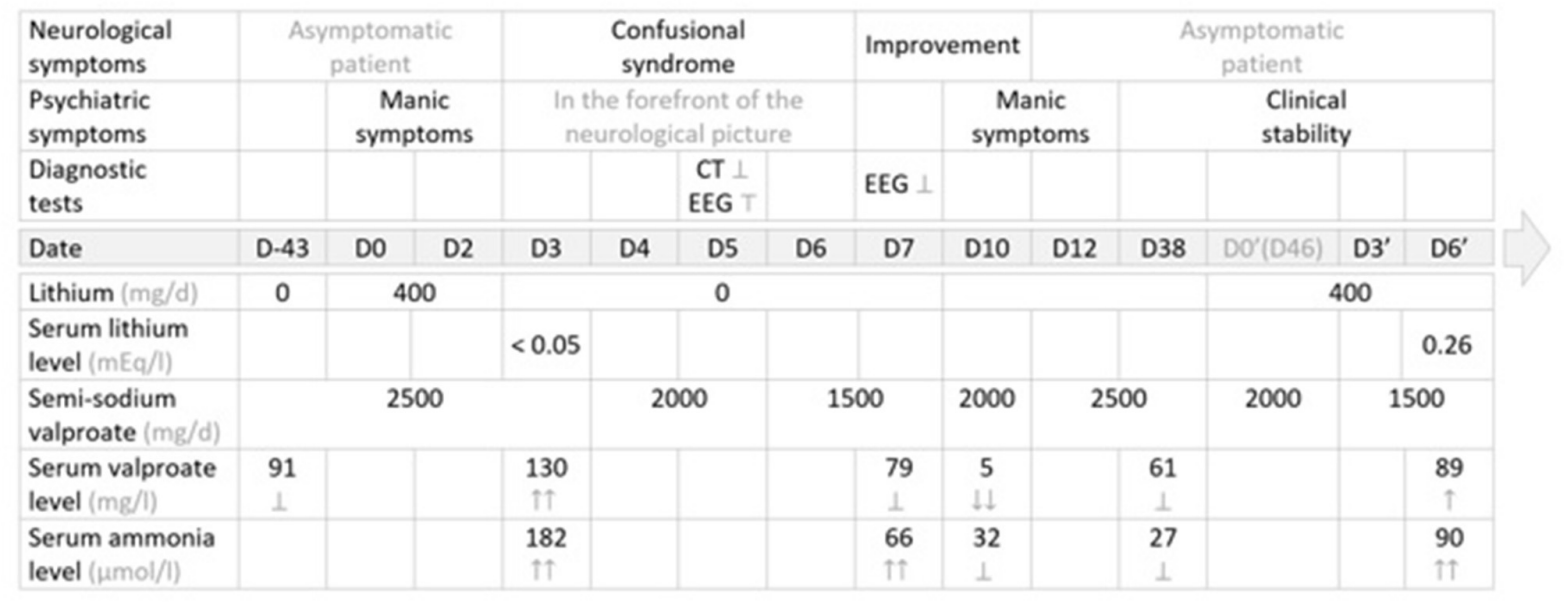
Management summary: therapeutic strategy, clinical course and diagnostic tests. EEG, electroencephalogram; ER, extended release; NA, not administered; N, normal values **→**; CT, computed tomography (brain) scan; Ṭ, abnormal; **?**, normal, within normal limits; **↑**, elevated value, within normal limits; **↑↑**, elevated value, above normal or supratherapeutic; **↓**, decreased value, within normal limits; **↓↓**, decreased value, below normal or subtherapeutic; ↕: discontinued; **→** Normal values: serum lithium level: *N* = (0.84–1.2) mg/l; serum valproate level: *N* = (41–100) mEg/l; serum ammonia level: *N* = (16–60) μmol/l.

The first administration of lithium salts was temporally related to an elevation of valproate serum levels, which was in turn associated with hyperammonemia and signs and symptoms of encephalopathy, which resolved after lithium discontinuation and progressive reduction of valproate semisodium dosage. The rechallenge of the combination long after the first episode then enabled a more objective examination of another increase in the serum valproate level, with more moderate hyperammonemia (without encephalopathy or associated *clinical* signs), concomitant with the re-initiation of lithium, despite a progressive decrease in the doses of valproate semisodium administered (Figure 1).

## Discussion

Several hypotheses have been put forward in the literature regarding the mechanisms of valproate-related HAE (15, 16). Valproate-related HA appears to be the result of a disturbance in the ammonia production/elimination balance, involving various metabolic pathways. In fact, in the liver, blockage of the urea cycle by the active valproate semisodium metabolites (valproyl-CoA) might be the source of an elimination defect of ammonia in the water-soluble form. To a lesser extent, in the kidneys, valproate semisodium might induce an increase in mitochondrial transport of glutamine and might stimulate the production of ammonia during the transformation of glutamine into glutamate (6, 17).

In addition to these inhibition mechanisms (N-acetylglutamate synthase) and enzyme induction (glutaminase), the effects of valproate semisodium on the metabolic pathways might also be related, one the one hand, to the production of neurotoxic derivatives of the drug [2-ene-valproate, from β-oxidation (18)] (19) and, one the other hand, to the spatial configuration of valproate semisodium which is similar to that of fatty acids (FAs). Because of this similarity, valproate semisodium and FAs might compete during transport (linked to carnitine), mitochondrial metabolization (competition with a β-oxidation enzyme and metabolic deviation of FAs toward an ω-oxidation pathway with an accumulation of hepatotoxic derivatives) and urinary elimination (minority: 0.1%) as valproyl-carnitine (6).

Therefore, a pre-existing carnitine deficiency (undernutrition, dialysis) could contribute to an elevated serum valproate level which is then maintained by the effects of valproate semisodium itself (6, 20). Consequently, L-carnitine supplementation has been suggested in case of HAE, and has shown an effect on the speed of serum ammonia normalization (not correlated to the severity of encephalopathy) but without proving its effectiveness on clinical outcomes (20, 21).

In addition to the neurotoxicity of valproate semisodium metabolites, valproate-induced HA could also be responsible for an alteration in astrocyte glutamatergic metabolism which causes cerebral edema and an increase in sensitivity to oxidative stress which is likely to contribute to the occurrence of encephalopathy (15, 18, 22).

Nevertheless, there is no systematic deterministic correlation between valproate semisodium, HA and encephalopathy. Not all reported cases of valproate-related encephalopathy are accompanied by HA (16, 23) and almost half (16–52%) of the patients on valproate semisodium could have asymptomatic HA (i.e. without the occurrence of HE) (15, 23) that might not require therapeutic adjustment. Among the risk factors identified, age (0–2 years), impaired renal or hepatic function, carnitine deficiency, urea cycle disorders (including ornithine transcarbamylase deficiency), and combination therapy [particularly antiepileptic drugs (24)] appear to be associated with valproate-related HAE (10). However, no association was found between the occurrence of these AEs and the duration of treatment, the history of valproate semisodium use, the dose administered, or the serum valproate level [no dose effect (15, 23)] (10).

Therefore, if previous treatment attempts or the duration of current treatment were not protective factors for this patient regarding urea cycle, in principle, he had none of the above-mentioned *clinical* risk factors either. In hindsight, however, this study lacks information on the *biological* features that could have altered the metabolic pathways. Genetic testing for polymorphisms for the cytochrome P450 or other genes involved in mitochondrial disease leading to hyperammonemia or deficit of urea cycle has not be performed, and the presence of any urea cycle defects was not systematically assessed (plasma amino acid dosages, as well as urinary levels of orotate regarding OTC deficiency). Yet, it should be noted that serum carnitine level was not measured, but that clinical and biological improvement was rapidly observed after therapeutic adjustment without L-carnitine supplementation. Consequently, *other* parameters could have contributed to the development of HAE several years after the initiation of valproate semisodium, several months after the last adjustment in the treatment dose, and 3 days after combination with a mood stabilizer in a patient who also receives antipsychotics and benzodiazepines, associated with a recent *unusual* consumption of toxic substance (isolated cannabis use).

### Cannabis

Pharmacokinetic studies conducted on the therapeutic form of cannabis [cannabidiol—indicated in the management of symptoms related to refractory spasticity in multiple sclerosis and in syndromic seizures in children (25)] have shown a risk of hepatocyte damage with elevated transaminases (not found in the patient) during concomitant use of cannabidiol and valproate semisodium, as well as an inhibitory effect on certain isoforms of uridine-5'-diphospho-glucuronosyltransferases (UGT) and cytochromes P450 (CYP). This interaction involved in particular UGT1A6, 1A9, and 2B7 [the major valproate semisodium metabolism pathway by glucuronidation (40%) (26)] and to a lesser extent CYP2B6, 2C9, and 2C19 [also involved in valproate semisodium metabolism (27)] with an associated risk of increased serum levels of drugs metabolized by these isoenzymes. Therefore, cannabis use at the time of the first therapeutic switch could probably have contributed to an increase in serum valproate level by enzymatic inhibition [*or even* competition for UGT 1A9 and 2B7 (25)]. However, it would be difficult to explain the elevated serum valproate level observed during the second switch by this mechanism, considering that the patient was no longer using cannabis during this period.

### Adjunctive Medicines

Although it has been suggested that “endogenous benzodiazepines” (the γ-aminobutyric acid pathway) contribute to the occurrence of hepatic encephalopathy (28), the role of concomitant use of benzodiazepines in the occurrence of valproate-related HAE (beyond an additional confusional effect) does not appear to be based on any tangible evidence (22). In addition, in this case, the doses of diazepam re-initiated on admission (20 mg/d) were being reduced at the time of onset of the symptoms (5 mg/d at the time of the first switch, 3 mg/d at the time of the rechallenge). Similarly, if the concomitant administration of antipsychotics is to be taken into account in the occurrence of valproate semisodium-related AEs, a particular concern is the risk of extrapyramidal syndromes (29, 30) which do not justify special systematic precautions when antipsychotics are co-prescribed (2). For the patient, monthly injections of paliperidone were administered 18 days before the first switch, and 6 days before the second, with no documented signs of intolerance, and the dose of levomepromazine administered on admission (identical at the time of the rechallenge: 300 mg/d) (day 46) had been reduced by a factor of 6 before the first switch (55 mg/d) without preventing the occurrence of HAE.

### Lithium

In order to maximize tolerability and reduce the occurrence of side effects when initiating *de novo* lithium therapy, current guidelines suggest that lithium should be administered in small divided doses, and that the dose should be titrated gradually (31–33). For this purpose, and due to the narrow therapeutic index of lithium, the mood stabilizer was initiated (D0 and D0') at 400 mg/d and no dose increase was carried out before the onset of symptoms that led to an anticipated measurement of serum drug levels (undetectable levels on D3) or before the routine therapeutic drug monitoring that was performed in an asymptomatic patient (infratherapeutic levels on D6'). Thus, despite the *apparent* temporal concordance between the initiation of lithium and the onset of HAE, neither the semiological and biological profile (isolated hyperammonemia) of the encephalopathy, nor the onset of symptoms on day 3 of initiation [given the half-life of the drug (34) and undetectable blood levels on D3] supported the hypothesis of lithium-induced encephalopathy, which was less likely in this context than a hypothesis of valproate-induced encephalopathy. However, the appearance of symptoms 3 days after the initiation of lithium and their resolution 5 days after it was discontinued (associated with the dosage reduction of valproate semisodium), in addition to a *direct* relationship, suggests the possibility of *mediation* by thium in the occurrence of valproate-related HAE.

### Valproate Semisodium + Lithium Combination

The causality criteria in pharmacovigilance (35) find an intrinsic imputability of level I5, “very likely” (combining a chronological score of C3 “very likely” with a semiological score of S2 “likely”), and an extrinsic imputability rated B2 in the context of an adverse effect “not recognized in standard publications.” In fact, despite frequent co-prescription of these two drugs (11), there is little data available on the co-prescription of valproate semisodium and lithium. To our knowledge, a specific search for a drug interaction when lithium and valproate semisodium are combined has only been the focus in one pharmacokinetic study (a prospective controlled crossover study against placebo) which showed no change in serum lithium level, but a “slight” increase in the serum valproate level (minimum and maximum concentrations and area under the plasma concentration-time curve) with a dual mood-stabilizing drug therapy (36). However, the AE profile was not noted to be altered in this study and the AEs documented since then appear to be more related to the *cumulative* toxicities of each drug than to an underlying drug interaction (37).

### Considerations on the Association

Although this case raises the question of a pharmacokinetic interaction, the practice of prescribing lithium salts in combination with valproic acid is a common therapeutic strategy, which has interested several studies among bipolar I patients. Indeed, despite a possible increase in reported adverse events (including gastrointestinal distress, tremor, cognitive impairment, and alopecia), the authors of a literature review acknowledged that their imputability to the association rather than to one of the two molecules was not established, and that the co-administration of lithium plus valproate had generally produced favorable results, with particular benefit in patients with manic, mixed, and rapid-cycling features (38). More recently, a naturalistic study concluded that bipolar I patients on lithium plus valproate showed greater improvement in mixed, anxiety, and psychotic symptoms than those on lithium monotherapy and even suggested that a serum lithium level albeit below the reference range seemed sufficient to maintain clinical efficacy when associated with valproate (39). In the same way, a multicentric study that focused on relapse prevention in bipolar I patients showed in turn a superiority of combination therapy over valproate monotherapy, with no difference regarding adverse events and tolerability between the treatment groups (11). Thus, it seems relevant to emphasize that co-prescription of lithium and valproate is supported by evidence of clinical efficacy (acute treatment and relapse prevention) as well as safety.

## Conclusion

This case study raises the question of a possible drug interaction between lithium and valproate, based on the observation of an increase in serum levels of valproate when co-prescribed with lithium, effects being notable as of the initiation of the mood stabilizer (below the therapeutic thresholds) with robust results on rechallenge of the dual therapy. At the same time, these observations support the hypothesis of an association between serum valproate level elevation, hyperammonemia and reversible encephalopathy in a patient with no documented history of valproate semisodium intolerance. Therefore, given the frequency of the combination in everyday psychiatric practice and the potential seriousness of the adverse effects reported, a more in-depth pharmacokinetic exploration would provide a better understanding of the mechanisms underlying these interactions, as well as enable specification of the risk factors associated with their occurrence and provide support for the benefit-risk balance associated with the prescription of these drugs.

## Data Availability Statement

The original contributions presented in the study are included in the article/supplementary material, further inquiries can be directed to the corresponding author/s.

## Ethics Statement

Written informed consent was obtained from the individual(s) for the publication of any potentially identifiable images or data included in this article.

## Author Contributions

AL: writing. EV, FM, AJ, and PB: revising. LR: revising and teaching. All authors contributed to the article and approved the submitted version.

## Conflict of Interest

The authors declare that the research was conducted in the absence of any commercial or financial relationships that could be construed as a potential conflict of interest.

## Publisher's Note

All claims expressed in this article are solely those of the authors and do not necessarily represent those of their affiliated organizations, or those of the publisher, the editors and the reviewers. Any product that may be evaluated in this article, or claim that may be made by its manufacturer, is not guaranteed or endorsed by the publisher.
